# The X-ray crystal structure of the N-terminal domain of Ssr4, a *Schizosaccharomyces pombe* chromatin-remodelling protein

**DOI:** 10.1107/S2053230X20015216

**Published:** 2020-11-25

**Authors:** Janet Newman, Tom Nebl, Huy Van, Thomas S. Peat

**Affiliations:** a Biomedical Program, CSIRO, 343 Royal Parade, Parkville, VIC 3052, Australia

**Keywords:** chromatin remodelling, SAD phasing, novel structure, *Schizosaccharomyces pombe*

## Abstract

Ssr4 is a yeast protein from *Schizosaccharomyces pombe* and is an essential part of the chromatin-remodelling [SWI/SNF and RSC (remodelling the structure of chromatin)] complexes found in *S. pombe*. These complexes (or their homologues) regulate gene expression in eukaryotic organisms, affecting a large number of genes both positively and negatively. The downstream effects are seen in development, and in humans have implications for disease such as cancer. The chromatin structure is altered by modifying the DNA–histone contacts, thus opening up or closing down sections of DNA to specific transcription factors that regulate the transcription of genes. The Ssr4 sequence has little homology to other sequences in the Protein Data Bank, so the structure was solved using an iodine derivative with SAD phasing. The structure of the N-terminal domain is an antiparallel β-sheet of seven strands with α-helices on one side and random coil on the other. The structure is significantly different to deposited structures and was used as a target in the most recent Critical Assessment of Techniques for Protein Structure Prediction (CASP; https://predictioncenter.org/) competition.

## Abstract 2

The N-terminal domain of Ssr4 from *S. pombe*, which is an integral protein in the SWI/SNF and RSC chromatin-remodelling complexes, has been crystallized and its structure has been solved using iodine as a phasing vehicle.

## 1. Introduction   

Chromatin-remodelling complexes are essential for life and are evolutionarily conserved. These complexes have recently been implicated in cancer progression, either owing to their intrinsic ability for tumour suppression or their ability to alter the expression of tumour-suppression gene products (Roberts & Orkin, 2004 ▸). Much of our understanding of the chromatin-remodelling process comes from studying the two complexes found in the yeast *Saccharomyces cerevisiae*: SWI/SNF and RSC. The structures of both of these multicomponent machines have recently been determined using cryo-EM (Han *et al.*, 2020 ▸; Wagner *et al.*, 2020 ▸). The yeast *Schizosaccharo­myces pombe* is sometimes considered to be a more relevant model system for metazoan biology (Monahan *et al.*, 2008 ▸), and also contains the chromatin-remodelling complexes SWI/SNF and RSC, but the *Schizosaccharomyces pombe* complexes have different subunit compositions to the *S. cerevisiae* complexes. Ssr4 (UniProt entry Q9P7Y0) is one of the proteins that is not conserved between the two organisms and is only found in *S. pombe*.

There are at least 12 components of the SWI/SNF global transcription-activator complex in *S. pombe* and these include at a minimum Arp9, Arp42, Snf5, Snf22, Snf30, Sbf59, Sol1, Ssr1, Ssr2, Ssr3, Ssr4 and Tfg3. Additionally, the RSC complex is composed of at least 13 components including Arp9, Arp42, Rsc1, Rsc4, Rsc7, Rsc9, Rsc58, Sfh1, Snf21, Ssr1, Ssr2, Ssr3 and Ssr4. These complexes interact with histones and histone-variant components of the eukaryotic chromatin. They regulate transcription by altering the contacts made between DNA and the histones that coat the DNA, therefore opening up or closing down the sections available for gene regulation. Typical modifications include the acetylation or methylation of various lysine residues found on histones, the phosphorylation of serines on the histones and the methylation of cytosine bases on the DNA (Strahl & Allis, 2000 ▸). Most of these modifications change the interactions between the histones and the DNA, which allows or denies the access of transcription factors and RNA polymerase to the promoter regions of the genome. On the flip side, deacetylases, de­methyl­ases and phosphatases generally support the silencing of genes and the compaction of the chromatin (McKnight *et al.*, 2015 ▸).

We were interested in characterizing some of the less well known components that had no structural homologues in the Protein Data Bank (PDB). An initial search of the PDB with the Ssr4 sequence confirmed that there were no homologous structures (by sequence), which made it of interest to us. This also meant that there was no molecular-replacement model for this protein structure, so *de novo* phasing would be required.

## 2. Materials and methods   

### 2.1. Protein production   

The *ssr4* gene from *S. pombe* (coding for 395 residues, lacking the N-terminal methionine) was ordered from GenScript and inserted into a pET vector with a six-His tag and TEV cleavage site at the N-terminus. The protein was expressed in *Escherichia coli* BL21(DE3) cells, which were induced by the addition of isopropyl β-d-1-thiogalacto­pyranoside to 1 m*M* at an OD_600_ of 0.6–0.8 and allowed to grow for a further 4–5 h at 28°C. The cells were pelleted and frozen for further use later. The frozen cell pellet was thawed, resuspended in TBSA (25 m*M* Tris pH 7.6, 135 m*M* NaCl, 2.7 m*M* KCl, 0.02% NaN_3_), sonicated and centrifuged at 14 000 rev min^−1^ in a Beckman JA25.5 rotor to remove cell debris, and the supernatant was applied onto a 5 ml Ni–NTA column. The column was loaded with TBSA plus an extra 150 m*M* NaCl plus 10 m*M* imidazole, washed extensively with the same buffer with 20 m*M* imidazole and the protein was then eluted by the addition of 250 m*M* imidazole in TBSA with 150 m*M* NaCl. The protein was concentrated and applied onto a Superdex 200 16/60 gel-filtration column pre-equilibrated in TBSA. The peak fraction from the size-exclusion column was concentrated to 5 mg ml^−1^ and stored at 4°C. Approximately 4.5 mg of the full-length protein was obtained from a 1 l culture. The initial protein sample ran as a 46 kDa band on an SDS–PAGE gel, but gels run after storage at 4°C showed a major band that ran at an apparent molecular mass of 25 kDa (Supplementary Fig. S1*a*). Trypsin (Sigma, catalogue No. T1426) was dissolved in water to 1 mg ml^−1^ and 10 µl aliquots were lyophilized in PCR tubes. The protein was treated with trypsin by adding 50–100 µl aliquots of the protein sample to the PCR tubes containing 10 µg lyophilized trypsin and incubating on ice for an hour or more. The protein was used after trypsin treatment with no further purification.

### 2.2. Differential scanning fluorimetry (DSF)   

The native and trypsin-treated proteins were characterized by DSF, where 0.30 µl of protein at 4 mg ml^−1^ was diluted into a final volume of 20 µl. 0.3 µl of a 1:20 dilution of SYPRO Orange dye (Sigma, catalogue No. S5692) was added and the temperature was ramped from 20 to 100°C in 0.5°C steps in a plate-based real-time PCR machine (Bio-Rad CFX-96; Supplementary Figs. S2*a* and S2*b*). The fluorescence curves were analysed with *Meltdown* (Rosa *et al.*, 2015 ▸). 13 different buffers ranging from pH 5 to 9, each at two NaCl concentrations (50 and 200 m*M*), were tested (Seabrook & Newman, 2013 ▸), along with the initial TBSA formulation.

### 2.3. Mass spectrometry (MS)   

Intact mass determination of Ssr4 preparations was achieved by LC-MS as described previously (Newman *et al.*, 2019 ▸). Briefly, native and partially trypsin-digested protein samples were spiked with formic acid (FA) to a final concentration of 0.1%(*v*/*v*) and separated by reverse-phase liquid chromatography on an UltiMate 3000 RSLCnano system (Thermo Fisher Scientific) fitted with a 150 × 4.6 mm, 5 µm particle-size, 300 Å pore-size PLRP-S column (Agilent). Proteins were eluted at a flow rate of 250 µl min^−1^ by applying a linear 30 min gradient from 0 to 80% solvent *B* [mobile phase *A*, 0.1%(*v*/*v*) FA; mobile phase *B*, 90%(*v*/*v*) acetonitrile/0.1%(*v*/*v*) FA] and ionized using an Apollo II electrospray ion source coupled to a MaXis II mass spectrometer (Bruker). High-resolution LC-MS data were analysed using the *Intact Mass* parsimonious charge-state deconvolution algorithm (Protein Metrics, USA; Supplementary Figs. S1*b*–S1*f*).

### 2.4. Crystallization   

Crystallization experiments were set up in SD2 sitting-drop plates (Molecular Dimensions, UK) at 8°C with droplets consisting of 200 nl protein solution plus 200 nl reservoir solution (1:1 ratio) with 50 µl reservoir solution in the wells. Some of the drops were microseeded in an effort to control nucleation and produce larger crystals. A seed stock was made by adding the contents of the reservoir and the crystal drop to a 1.5 ml centrifuge tube, adding a few (5–10) 0.4 mm glass beads and vortexing for a minute. Seeded drops contained 200 nl protein, 180 nl reservoir and 20 nl of the seed stock (Newman *et al.*, 2008 ▸). The protein concentration was 5 mg ml^−1^ in TBSA. Three different protein samples were used in crystallization: protein that had been purified and stored at 4°C, protein that had been purified and treated with trypsin and protein that had been treated with extra protease inhibitors. For this, freshly purified protein was treated with a 1:100 volume ratio of 100× Roche cOmplete protease-inhibitor cocktail (one tablet dissolved in 0.5 ml 100 m*M* EDTA).

**Figure 1:**
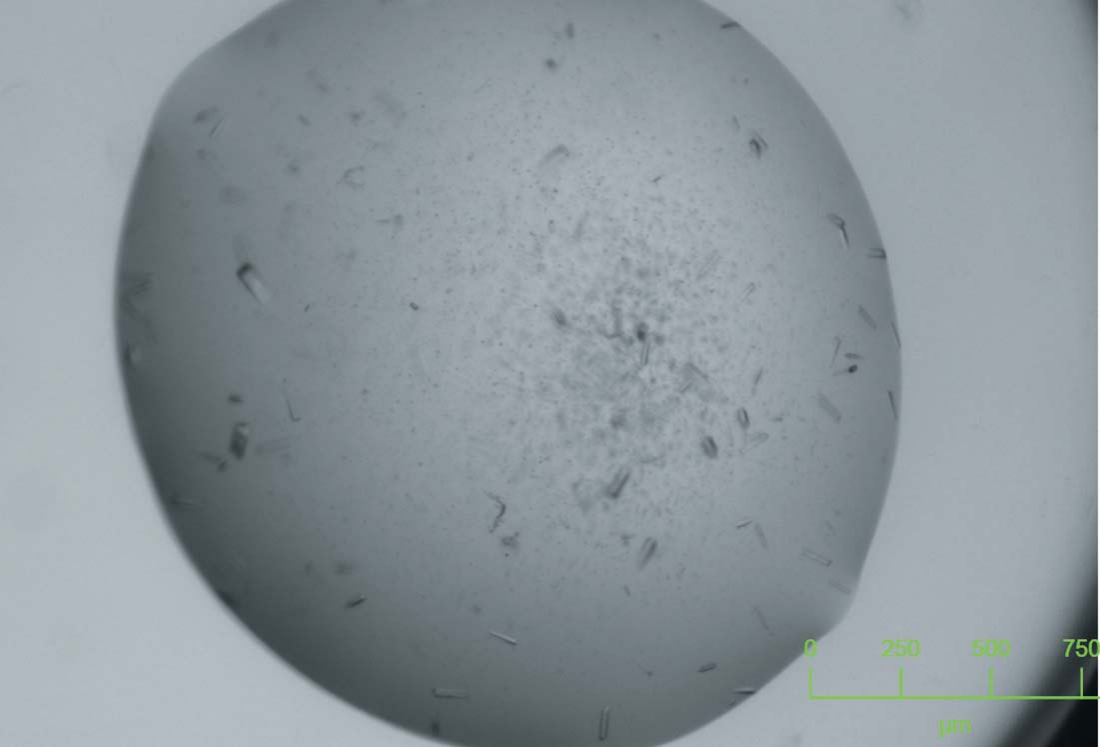
Crystals of Ssr4.

Rod-shaped crystals (Fig. 1 ▸) grew from both native protein stored at 4°C and trypsin-treated protein using reservoir conditions consisting of 1.5–1.9 *M* ammonium sulfate, 0.7–12% dioxane and either 100 m*M* MES, 100 m*M* bis-tris or 10%(*v*/*v*) malate–MES–Tris buffer at a pH between 5.5 and 5.8. Crystals appeared after a week and grew to full size (30 × 30 × 120 µm) over the course of a month. No indication of crystal formation was observed for the inhibitor cocktail-treated protein even after setting up ten 96-condition screens at two different protein concentrations.

### 2.5. Data collection and processing   

Glycerol was added to the reservoir to give a final concentration of 20%; crystals were cryoprotected by adding 2 µl of the glycerol-doped reservoir to the drop with crystals. Iodine was introduced into the crystals by adding a tiny crystal of solid I_2_ to the reservoir solution (along with the glycerol) and this was used to briefly soak the crystals. Crystals were removed using a 50 µm MiTeGen Mylar loop and the crystals were cryocooled by plunging them into liquid nitrogen. Data were collected on the MX2 microfocus beamline at the Australian Synchrotron. 360° of data were obtained with the wavelength set to 8007.8 eV for the iodine-treated crystals. Data sets were collected at 13 600 eV from the native crystals. The data were processed with *XDS* (Kabsch, 2010 ▸) and scaled with *AIMLESS* (Evans & Murshudov, 2013 ▸). Xenon, bromine and native sulfur SAD data sets were also collected at appropriate energies to obtain the maximum anomalous signal given the constraints of the beamline.

### 2.6. Structure solution and refinement   

**Figure 2:**
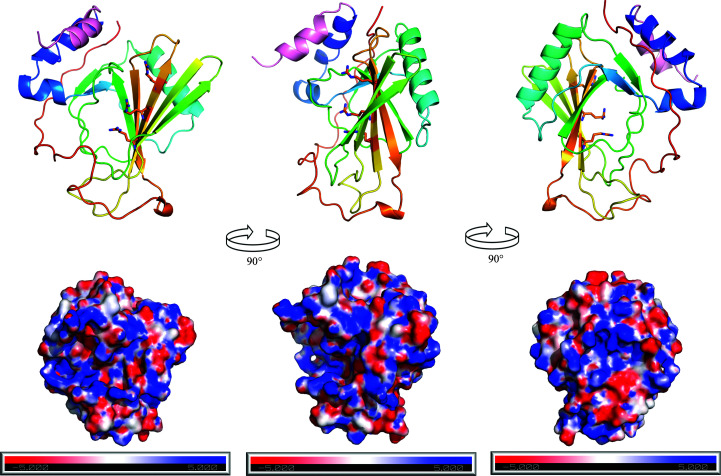
Ribbon and electrostatic views of Ssr4. The top panel shows three views of Ssr4 as a cartoon ribbon, highlighting the secondary structure of the protein. The N-terminal His tag is coloured pink to distinguish it from the rest of the native structure, which uses the standard Jones colouring of blue for the N-­terminus to red at the C-terminus. The rotations between figures are an approximately 90°, making the leftmost and rightmost images rotated by 180°. Also shown in sticks are the three arginine residues along β6. Below, in the same orientation, is the electrostatic potential calculated by *APBS*. This figure was made using *PyMOL* (version 1.8; Schrödinger) and *Gimp* (https://www.gimp.org/).

**Figure 3:**
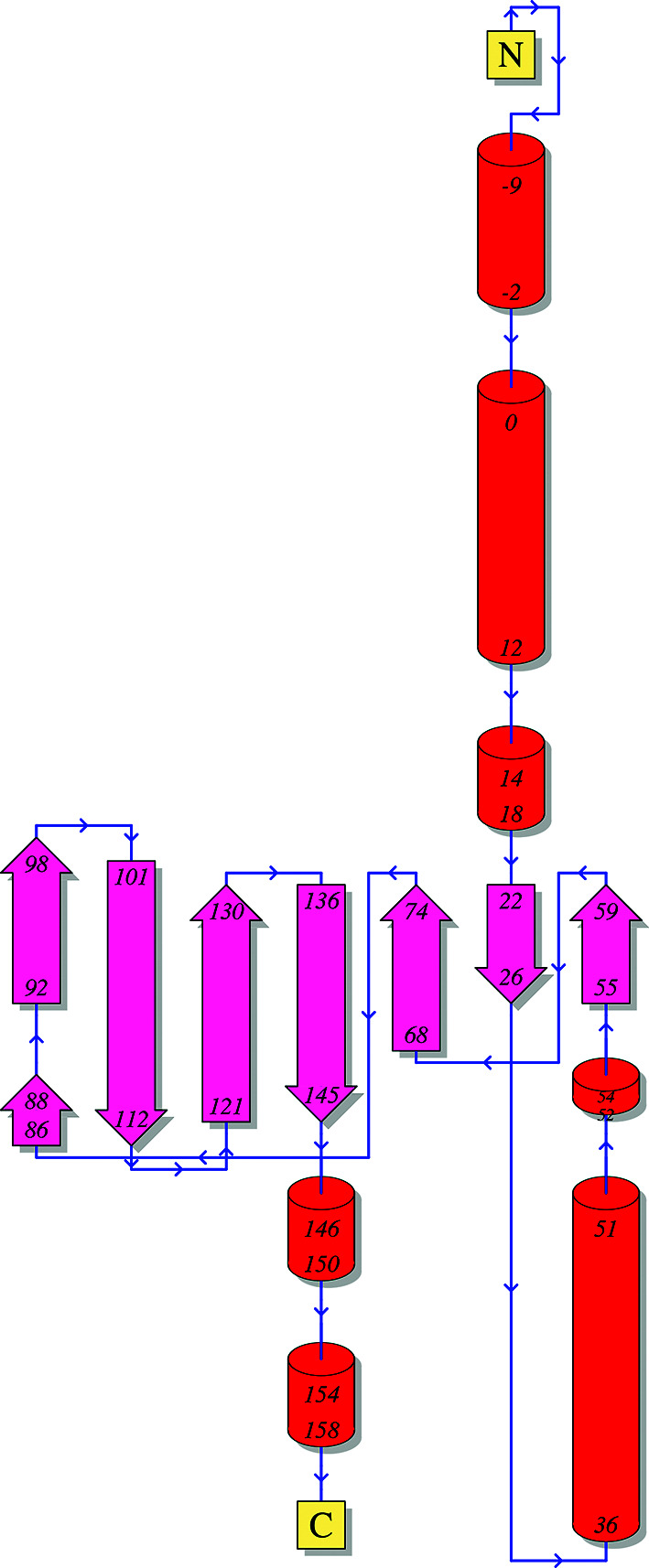
Topology of the Ssr4 protein with the N-terminal tag included as determined by *PDBSum*.

The structure was initially solved with *Auto-Rickshaw* (Panjikar *et al.*, 2005 ▸) using the iodine-soaked crystal data set in which two iodine sites had been located. The *Buccaneer* (Cowtan, 2006 ▸) model was then used as a starting point for manual rebuilding in *Coot* (Emsley *et al.*, 2010 ▸) and refinement performed in *REFMAC* (Murshudov *et al.*, 2011 ▸). *MolProbity* (Chen *et al.*, 2010 ▸) was used to determine various quality measures of the structure, including the Ramachandran statistics listed in Table 1 ▸. Only the N-terminal half of the protein was seen in the crystal structure (Fig. 2 ▸); a topology diagram of the structure from *PDBSum* (Laskowski *et al.*, 2018 ▸) is shown in Fig. 3 ▸.

## 3. Results and discussion   

The full-length protein was expressed and initially purified, but it was cleaved during storage to leave just the N-terminal domain. The initial purification showed a major band at the expected weight of 46 kDa, but there were numerous minor contaminating bands, one of which we assume was a protease. Although the protein was cleaved by some contaminant, the addition of trypsin to the (cleaved) protein sample increased the number of crystals that were formed. The crystals themselves were orthorhombic, but with very similar *b* and *c* axes (see Table 1 ▸), so that autoprocessing would sometimes misassign the space group as tetragonal.

Thermal melt curves were obtained for both the sample treated with protease inhibitor and the trypsin-treated sample. The protease-inhibited sample showed a high initial fluorescence in all buffer/salt combinations, but showed a clear melt transition, with a *T*_m_ of 58.0 ± 0.1°C, in TBSA. The trypsin-treated sample showed flatter pre-transition curves and gave a *T*_m_ of 60.3 ± 0.1°C. This indicated that there might be a portion of the protein that was disordered under the conditions tested. The *Meltdown* reports are shown in Supplementary Fig. S2.

In the MS analysis, the protease-inhibited sample eluted as a single peak with a retention time of ∼15.4 min, with a major mass of 46 256 Da matching full-length Ssr4 with the loss of the N-terminal methionine. Accurate mass analysis is consistent with the loss of the N-terminal methionine (−131 Da), the presence of a single disulfide bond (−2 Da) and partial (phospho)gluconoylation of the His tag (+178/+258 Da), and some C-terminal clipping at Glu377. The partially trypsin-digested SSR4 sample produced a complex ion chromatogram showing two prominent peaks. Peak 1 at a retention time of ∼11.6 min contained a major mass of ∼14 172.3 Da, which was most likely to correspond to a C-terminal Gly298–Arg411 fragment, and peak 2 at 15.8 min contained a major mass of 22 401.6 Da, matching an N-terminal Gly2–Lys199 fragment with partial (phospho)gluconoylation of the His tag (+178/+258 Da) (see Supplementary Figs. S1*b*–S1*f*).

The Ssr4 structure was solved using a crystal that had been soaked with iodine and which showed two major peaks for I atoms in the electron density (about 40σ and 30σ above the background), with three more peaks (12σ, 11σ and 5.5σ) that corresponded to either low-occupancy I or Cl atoms (some of these peaks are also seen in non-iodine-soaked structures). *Auto-Rickshaw* (Panjikar *et al.*, 2005 ▸) was used to find the iodines, phase the data and build the initial structure via *Buccaneer* (Cowtan, 2006 ▸). After manual rebuilding and refinement, this initial structure was then used to phase a slightly higher resolution native data set (using *Phaser*; McCoy *et al.*, 2007 ▸), and manual rebuilding using *Coot* (Emsley *et al.*, 2010 ▸) gave a more complete structure, which was again refined using *REFMAC* (Murshudov *et al.*, 2011 ▸) (see Table 1 ▸). The initial structure was also used to phase the sulfur SAD data using *Phaser* to determine whether these highly redundant data offered any additional aspects on the structure and for *post hoc* analysis. Density was seen for residues extending from the N-terminal tag to residue 179/180 of the Ssr4 protein in all three structures.

The structure of the Ssr4 N-terminal domain (Figs. 2 ▸ and 3 ▸) starts with a short helix (α1; residues 2–12), followed by another helix (α2; residues 15–20), a short β-strand (β1; residues 22–25) and then an unstructured coil that leads to a long helix (α3; residues 37–51). This then leads to another short strand (β2; residues 56–59), a loop to strand β3 (residues 68–73), a long loop to strand β4 (residues 92–98), a short turn to strand β5 (residues 101–108), a medium-size loop to strand β6 (the longest β-strand; residues 122–130) and a short loop to strand β7 (residues 137–144). The rest of the structure, residues 145–179/180, is mostly a long unstructured coil with two single-turn helical sections. The seven β-strands form an antiparallel sheet which is mostly covered on one side by the long helix (α3; residues 37–51), and on the other side of the β-sheet is a hole which has unstructured coils on the other side. Side chains fill this hole. It is interesting to note that the long β-strand β6 (residues 122–130) has a set of arginine residues all oriented in one direction emanating from it (Arg124, Arg126 and Arg128). We hypothesize that trypsin cleaves the protein shortly after the last residue modelled (Glu180), as the sequence at this point is Glu-Pro-Lys-Lys. *PDBeFold* (Krissinel & Henrick, 2004 ▸) was used to compare the Ssr4 structure with all other structures in the PDB. Two different structures of LytM (PDB entries 4zyb and 4bh5, from *S. aureus* and *E. coli*, respectively; Grabowska *et al.*, 2015 ▸; Peters *et al.*, 2013 ▸) can be aligned with an r.m.s.d. of 2.9–3.0 Å over 60–70 residues (*Q*-scores of 0.09–0.10), and PDB entry 5j6p (C. Shao, C. Wang, M. Zhang, C., Zhang & J. Zang, unpublished work), another *S. pombe* protein (Mis18) can be aligned with an r.m.s.d. of 4.1 Å over 67 residues. The aligned residues are parts of the β-sheet in both cases, with the rest of the protein not aligning at all. This suggests that this domain of SSR4 adopts a fold that is not currently represented in the public PDB. For this reason it was submitted to CASP14, with the results of the modelling to be presented in the future.

The N-terminal tag used for purification forms a helix from residues −9 to −2 and there is a break in the density in two of the three structures where the residues Ser1 and Ala2 should reside (Ser1 is the last residue of the tag and Ala2 is the first residue of native Ssr4). These two residues were modelled into one of the models as there is some weak density in the iodine-phased data, with a break at Gly0. The helical part of the tag is packed against the N-terminal α1 helix of the Ssr4 protein body (Fig. 2 ▸). The density becomes thin before the third histidine of the His tag, with four residues (MGHH) missing from the N-terminus of the tag.

We calculated the electrostatic potential of Ssr4 using *APBS* (Baker *et al.*, 2001 ▸; see Fig. 2 ▸). There is no clear face of potential that might signal specific binding to DNA or histones, for example. Further studies will be required to determine how Ssr4 interacts in the SWI/SNF and RSC complexes and what its role might be in these chromatin-remodelling complexes.

The structure was determined using the anomalous signal from the iodine-soaked crystals, although previous data sets using xenon, bromine and sulfur as potential phasing vehicles had been collected to relatively high resolution and with good statistics prior to obtaining the iodine data set. We observe four well ordered methionines (with one more that is slightly less ordered) and two cysteines in the structure that should contribute to an anomalous sulfur signal for a protein that is less than 200 amino acids in length, and the data extended to 2.1 Å resolution with about 50-fold redundancy/multiplicity on average for the anomalous data (100-fold for the full data set treating the Friedel mates as equivalent). Despite the good statistics and observable signal, the peaks found were weak (only about 5σ) and the structure could not be solved after trying several different software packages (*Auto-Rickshaw*, *CRANK*2, *Phenix AutoSolve* anad *SHELX*; Panjikar *et al.*, 2005 ▸; Skubák & Pannu, 2013 ▸; Liebschner *et al.*, 2019 ▸; Terwilliger *et al.*, 2009 ▸; Sheldrick, 2015 ▸).

We suspect that the trypsin neatened the C-terminus left by the contaminating protease to create a crystallizable sample: the residues C-terminal to those seen in the structure are PKK, which correspond to trypsin sites at residues 182/183 in the full-length protein. Once it was recognized that the protein sample set up in crystallization trials had been cleaved, a new preparation was made and special care was taken to prevent proteolysis. This sample did not yield crystals. However, trypsin treatment of freshly prepared protein did yield the familiar rod-shaped crystals. In the LC-MS results of the trypsin-treated protein, peak 1 matches a C-terminal tryptic fragment corresponding to a predicted globular Ssr4 domain Gly298–Arg411 and peak 2 matches a large N-terminal tryptic fragment corresponding to a predicted globular Ssr4 domain Gly2–Lys199 (the numbering in these cases refers to the Ssr4 protein with an N-terminal His tag and TEV site). LC-MS analysis of the native protein showed some clipping of the C-terminal region of the protein, suggesting that the C-terminal region, although folded, is less stable than the N-terminal fragment that crystallized. The central region is predicted to be intrinsically unstructured and is probably rapidly proteolysed.

Most of the tag sequence, HHHHGTENLYFQGS, has clear electron density and forms a helix that packs nicely against the N-terminal domain of Ssr4; thus, the first 14 residues modelled are not native to Ssr4 (pink helix in Fig. 2 ▸).

As there were no homologous structures in the PDB which might serve as a molecular-replacement model for this protein structure, we attempted to use several different methods for *de novo* phasing: xenon, bromine and iodine soaks and native sulfur SAD phasing. Even after soaking, most of the crystals diffracted to beyond 2.5 Å resolution and complete data sets were acquired. In the case of the sulfur SAD data sets, multiple data sets were acquired, and these were sufficiently isomorphous to give a merged data set with 100-fold total (50-fold anomalous) redundancy/multiplicity in the data. Despite the high redundancy and the reasonably high resolution (complete data extended to 2.1 Å resolution with reasonable statistics), there was insufficient sulfur signal to successfully phase the structure. Although the sulfur, bromine and iodine data sets all showed some anomalous signal in the scaled statistics, only the iodine data set had sufficient signal to solve the structure.

## 4. Conclusion   

We have crystallized and solved the structure of the N-terminal domain (up to residue ∼180 of 395) of Ssr4 using an iodine derivative. This structure does not align well with any other structure in the public PDB; we thus considered it to be ‘novel’ and it was submitted to CASP for this reason.

## Supplementary Material

PDB reference: N-terminal domain of Ssr4, native, 7k7w

PDB reference: iodine derivative, 7k7v

PDB reference: sulfur SAD data, 7k82

Supplementary Figures. DOI: 10.1107/S2053230X20015216/va5037sup1.pdf

## Figures and Tables

**Table 1:** Data-collection and processing statistics Values in parentheses are for the outer shell.

	Native	Iodine derivative	Sulfur SAD
PDB code	7k7w	7k7v	7k82
Data collection			
Space group	*P*2_1_2_1_2_1_	*P*2_1_2_1_2_1_	*P*2_1_2_1_2_1_
Wavelength (Å)	0.911684 [13600 eV]	1.548300 [8007.8 eV]	1.548600 [8006.2 eV]
*a*, *b*, *c* (Å)	50.10, 67.51, 67.46	50.16, 67.50, 67.53	50.34, 68.25, 67.78
Resolution (Å)	47.7–1.77 (1.81–1.77)	40.3–1.88 (1.92–1.88)	40.4–2.10 (2.16–2.10)
Completeness (%)	100 (100)	98.3 (78.3)	99.9 (99.6)
*R*_merge_	0.169 (2.445)	0.161 (1.246)	0.355 (4.755)
*R*_p.i.m._	0.047 (0.893)	0.046 (0.432)	0.036 (0.469)
CC_1/2_	0.998 (0.423)	0.997 (0.488)	0.999 (0.753)
Mean *I*/σ(*I*)	10.9 (1.1)	13.5 (2.2)	18.3 (1.7)
No. of unique reflections	23002 (1297)	18912 (976)	14204 (1128)
Multiplicity	13.5 (8.0)	13.0 (8.9)	97.3 (101.0)
Mosaicity (°)	0.12	0.22	0.42
Anomalous completeness (%)		97.5 (72.2)	100 (99.6)
Anomalous multiplicity		6.9 (4.9)	52.3 (52.7)
CC_anom_ (overall)		0.208	0.186
RCR_anom_ (overall)		1.235	1.207
No. of I or S atoms		2	7
Refinement			
Resolution (Å)	47.7–1.77	40.3–1.88	40.5–2.10
*R*_work_ (%)	17.7	16.8	19.2
*R*_free_ (%)	20.6	20.4	22.2
No. of atoms (total)	1708	1649	1612
No. of protein atoms	1557	1548	1547
No. of waters	138	89	58
Mean *B* value overall (Å^2^)	21.7	24.3	38.1
Protein *B* value (Å^2^)	22.5	25.6	40.4
Water *B* value (Å^2^)	31.8	31.6	41.9
R.m.s.d., bond lengths (Å)	0.012	0.012	0.010
R.m.s.d., angles (°)	1.632	1.625	1.534
Ramachandran analysis†			
Favoured	180	185	182
Outliers	1	1	1

† Ramachandran analysis from *MolProbity*.
