# Supplementary material for: The X-ray crystal structure of the N-terminal domain of Ssr4, a Schizosaccharomyces pombe chromatin-remodelling protein
Source: Acta Crystallogr F Struct Biol Commun. 2020 Nov 25;76(Pt 12):583–9. doi: 10.1107/S2053230X20015216 (PMC7716260; doi:10.1107/S2053230X20015216)
Supplement: Supplementary file 1 [file f-76-00583-sup1.pdf]

Acta Cryst F

STRUCTURAL BIOLOGY COMMUNICATIONS

Volume 76 (2020)

Supporting information for article:

The X-ray crystal structure of the N-terminal domain of Ssr4, a *Schizosaccharomyces pombe* chromatin-remodelling protein

Janet Newman, Tom Nebl, Huy Van and Thomas S. Peat

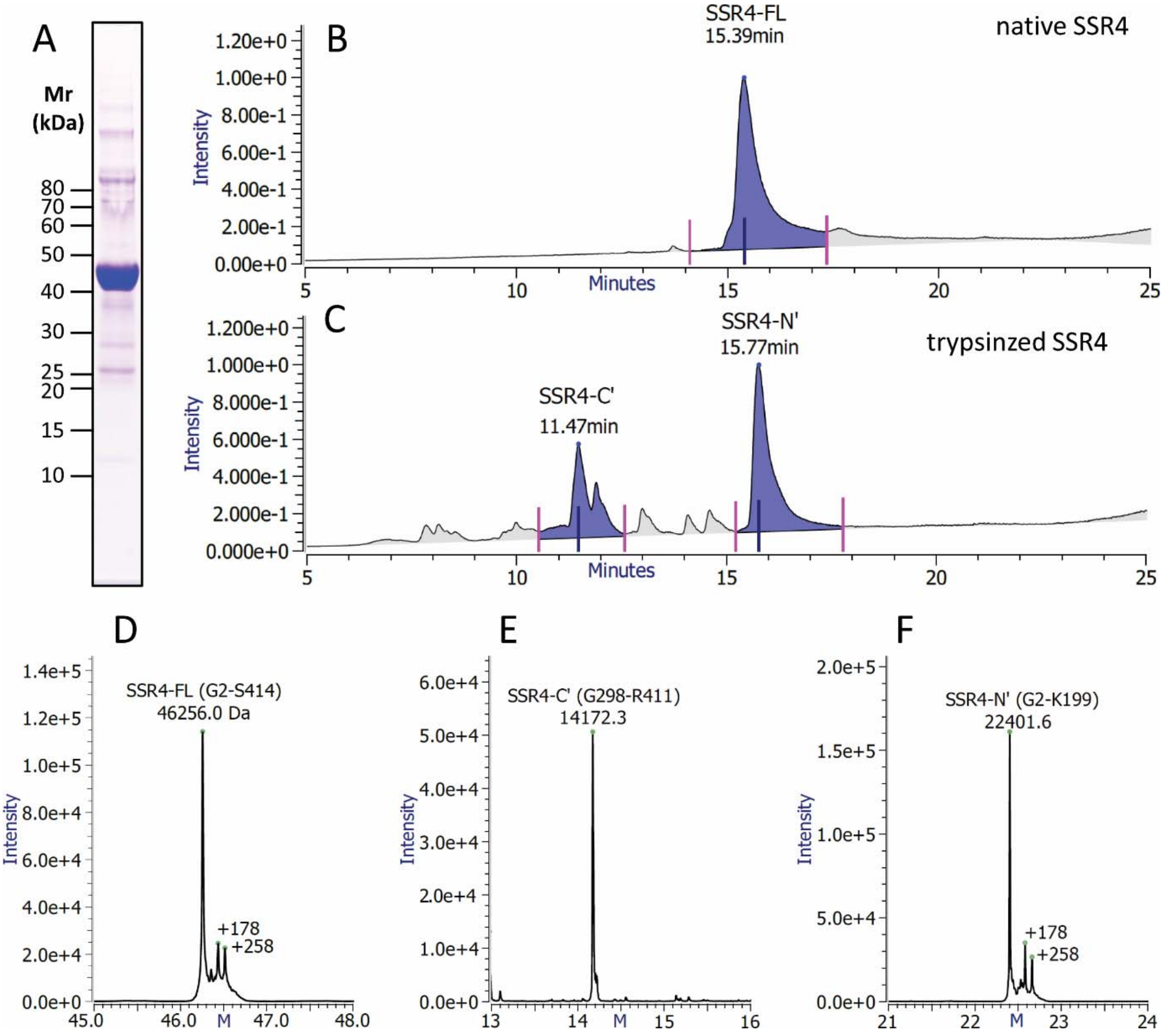

Figure S1 SDS-PAGE and LC-MS analysis of purified, recombinant SSR4 expressed in *E. coli.* Coomassie-stained SDS-PAGE analysis showed the major band at an apparent MW of ~46 kDa (**A**; arrow). Intact mass analysis by LC-MS of purified SSR4 treated with protease inhibitors showed a single peak at RT~15.4 min with a major mass of 46,256 Da matching full-length SSR4 (**B, D**; SSR-FL). After partial trypsin digestion, the SSR4 sample produced a complex ion chromatogram showing two prominent peaks (**C**). Peak 1 at RT ~11.6 min contained a major mass of ~14,172.3 Da most likely corresponding to a C-terminal G298-R411 fragment (**E**, SSR-C'), and Peak 2 at ~15.8 min contained a major mass of 22,401.6 Da matching a N-terminal G2-K199 fragment (**F**; SSR-N'). Accurate mass analysis is consistent with the loss of N-terminal methionine (-132Da) and partial (phospho)gluconoylation of the His-tag (+178/+258 Da) of SSR-FL and SSR-N' isoforms (**D, F**).

Figure S2 DSF Meltdown curves for the intact (46 kDa) and cleaved (25 kDa) SSR4 protein samples.
